# Airflow and dynamic circumference of abdomen and thorax for adults at varied continuous positive airway pressure ventilation settings and breath rates

**DOI:** 10.1038/s41597-023-02326-5

**Published:** 2023-07-22

**Authors:** Ella F. S. Guy, Jennifer L. Knopp, Theodore Lerios, J. Geoffrey Chase

**Affiliations:** Department of Mechanical Engineering, University of Canterbury, Christchurch, New Zealand

**Keywords:** Computational biology and bioinformatics, Medical research, Biomedical engineering

## Abstract

Continuous positive airway pressure (CPAP) ventilation is a commonly prescribed respiratory therapy providing positive end-expiratory pressure (PEEP) to assist breathing and prevent airway collapse. Setting PEEP is highly debated and it is thus primarily titrated based on symptoms of excessive or insufficient support. However, titration periods are clinician intensive and can result in barotrauma or under-oxygenation during the process. Developing model-based methods to more efficiently personalise CPAP therapy based on patient-specific response requires clinical data of lung/CPAP interactions. To this end, a trial was conducted to establish a dataset of healthy subjects lung/CPAP interaction. Pressure, flow, and tidal volume were recorded alongside secondary measures of dynamic chest and abdominal circumference, to better validate model outcomes and assess breathing modes, muscular recruitment, and effort. N = 30 subjects (15 male; 15 female) were included. Self-reported asthmatics and smokers/vapers were included, offering a preliminary assessment of any potential differences in response to CPAP from lung stiffness changes in these scenarios. Additional demographics associated with lung function (sex, age, height, and weight) were also recorded.

## Background & Summary

The burden of chronic respiratory diseases is increasing with increases in air pollution and obesity rates, alongside global mobility and transmission of disease^1–7^. Currently, diagnosis and treatment of respiratory disease rely predominantly on clinical judgement and tests which can only be conducted in clinical settings^8–15^. Hence, the capacity for diagnosis and treatment is limited and only capable of processing those with advanced disease. Targeting chronic disease with early diagnosis to prevent or slow disease progression, and thus reduce social and economic burden, would require increased testing capacity which is not currently economically feasible with many health systems already at/over capacity^16–19^.

Developing the capabilities of at-home respiratory monitoring would increase testing capacity and enable more effective remote monitoring and care. Significantly improved at-home testing would also reduce the burden of in-hospital testing on the healthcare system. For at-home testing to be implemented in an equivalent capacity to clinical testing requires robust modelling and model-based monitoring systems, able to identify clinically relevant metrics. The first step is high quality respiratory datasets to develop and test these methods.

Biomechanically, inspiration is driven by a decrease in pleural pressure, creating a pressure gradient between atmospheric pressure at the oral/nasal orifices and driving flow into the lungs through the respiratory tract^20,21^. Decreased pleural pressure is generated by expansion of the pleural space, predominantly driven by the descension of the diaphragm^20,21^. Additionally, intercostal muscle contraction raises the ribcage, which contributes to plural expansion^20,21^. Expiration is considered predominantly passive during normal breathing modes. However, it can be actively produced in cases of increased respiratory demand/load by eccentric diaphragm motion forcefully decreasing pleural volume and expelling air from the lungs^20,21^.

Diagnostically, chronic respiratory diseases are classified by the composition and location of obstructive and restrictive abnormalities^8,22^. Obstructive abnormalities act to occlude airways through constriction, inflammation, or collapse and cause increased airway resistance^8,23,24^. Restrictive abnormalities can be intrinsic and stiffen lung tissue by scaring/fibrosis or extrinsic and stiffen the thorax in cases of severe burns and obesity^3,8,25–27^. Clinically, the potential presence of abnormalities is primarily indicated by observed changes to breath patterns, palpation, percussion, auscultation and oximetry^8,28–30^.

Continuous positive airway pressure (CPAP) ventilation is a common respiratory therapy in both clinical and home settings^31,32^. CPAP provides a set positive end-expiratory pressure (PEEP), which reduces the patient work/effort required to generate inhalation by increasing the effective pressure gradient from expansion^33^. PEEP also maintains airway integrity, which can be impacted in cases such as obstructive sleep apnoea (OSA), preventing airway collapse^31,33,34^. The outlined trial was conducted to include a range of tested PEEP settings to understand the interaction between lung mechanics and CPAP using healthy subjects.

**Fig. 1:**
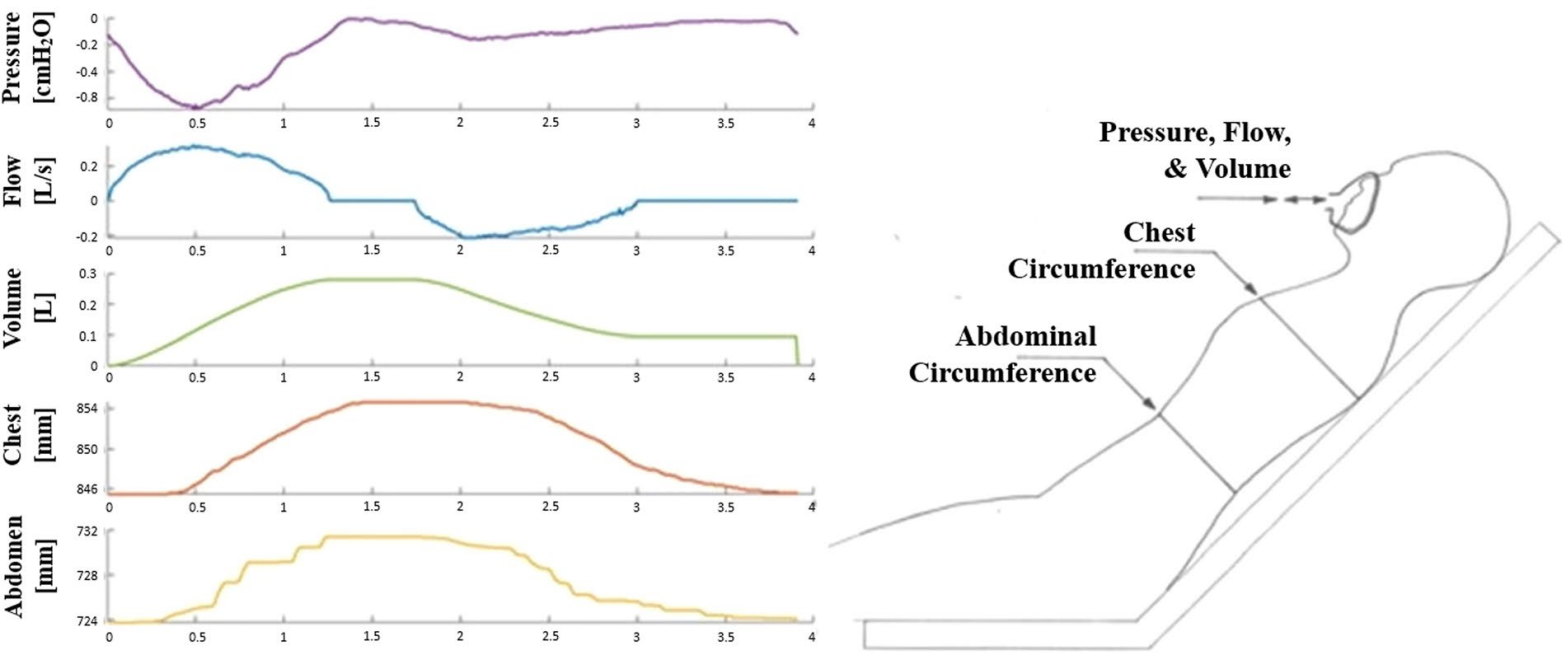
Data measurement illustration. The subject’s were semi-prone with abdominal circumference taken at the waist, chest circumference taken at the armpit level, and Pressure and Flow measured at the mask interface. An example of this collected data is illustrated for a single breath.

Pressure and flow data was recorded at the CPAP mask interface (mouth/nose orifice), from which tidal volumes were computed (Fig. 1). To elucidate thoracic and abdominal breathing modes circumferential monitoring was included in this trial (Fig. 1). These measures were taken at both chest and abdominal levels to better differentiate breathing modes (Fig. 1)^35^. This data also provides a second measurement set to validate and inform pressure and flow-based modelling.

The purpose of this dataset is to inform the development of model-based methods of respiratory assessment and treatment setting protocols. To personalise and optimise remote respiratory care, model-based methods must be developed to assess respiratory function including obstructive and restrictive abnormalities, signs of distress, and response to non-invasive mechanical ventilation (i.e. CPAP). Model-based methods have been used to identify patient effort, lung elastance, and airway resistance, with and without MV support^36–45^. This trial specifically examines the impact of CPAP on healthy lungs and respiration to capture the fundamental dynamics. Dynamic circumference measurements provide further information about muscular contributions to effort and different breathing modes.

## Methods

Ethical consent for the trial was granted by the Human Research Ethics Committee (HREC) at the University of Canterbury (HEC 2020/14/LR). Participants consented to the open publication of their de-identified data.

### Experimental design

The trial was designed for a cohort of N = 30 subjects, with an even split of males (15) and females (15), and including self-reported asthmatics and smokers. People with heart conditions or other serious medical conditions were excluded from participation. Table 1 outlines the trial procedure proposed to capture data from three breath rates (panting, normal, deep breathing) over three PEEP levels (ZEEP (zero end-expiratory pressure), 4 and 8 cmH_2_O).

**Table 1:** Trial schedule.

Investigator Procedure	Trial Sequence (PEEP and breath type)	Time
Preliminary Requirements: Explain procedure, consent forms, and demographic data survey		
Setup: Fit mask, circuitry, and monitoring system		
Instruct the patient to breathe normally and record data	ZEEP normal	65
Instruct the patient to pant as fast as they can and record data	ZEEP panting	35
	Recovery	Subject-determined
Instruct the patient to breathe as deeply as they can and record data	ZEEP deep	65
Turn on CPAP (4cmH2O)	Recovery	Subject-determined
Instruct the patient to breathe normally and record data	4 cmH_2_O normal	65
Instruct the patient to pant as fast as they can and record data	4 cmH_2_O panting	35
	Recovery	Subject-determined
Instruct the patient to breathe as deeply as they can and record data	4 cmH_2_O deep	65
Change PEEP to 8cmH2O	Recovery	Subject-determined
Instruct the patient to breathe normally and record data	8 cmH_2_O normal	65
Instruct the patient to pant as fast as they can and record data	8 cmH_2_O panting	35
	Recovery	Subject-determined
Instruct the patient to breathe as deeply as they can and record data	8 cmH_2_O deep	65
Pack up: Turn off CPAP and remove the mask, circuitry, and monitoring system	Recovery	Subject-determined

Breathing was un-cued (without a metronome) to capture as many breath modes as possible within the cohort in response to verbal instructions to pant as fast as possible, breathe normally, and breathe as deeply as possible. The trial was designed to include 4cmH_2_O PEEP, as it is a commonly prescribed initial or minimum PEEP level for patients during CPAP therapy^46–48^. A control level at ZEEP (PEEP = 0 cmH_2_O) was chosen to capture natural unassisted mechanics. A higher support level was subsequently set to 8cmH_2_O to provide a uniform level of increased support. All breathing rates were assessed at each PEEP level, with subject-determined breaks between each to mitigate fatigue.

Overall, the trial was designed to capture a broad range of mechanics to inform the design of more extensive trials aimed at improving the prescription of CPAP therapy, which currently has varied efficacy and adherence across different demographics^49,50^.

At the start of the trial, prior to obtaining informed consent, the researcher described the procedure to the subject, and provided them with a written summary. After consent was obtained, demographic data was collected using a questionnaire completed at the start of the trial, measurement equipment was made available for height and weight. Subsequently, a CPAP mask was fitted to the patient and the respiratory circuitry was connected.

### Data acquisition

**Fig. 2:**
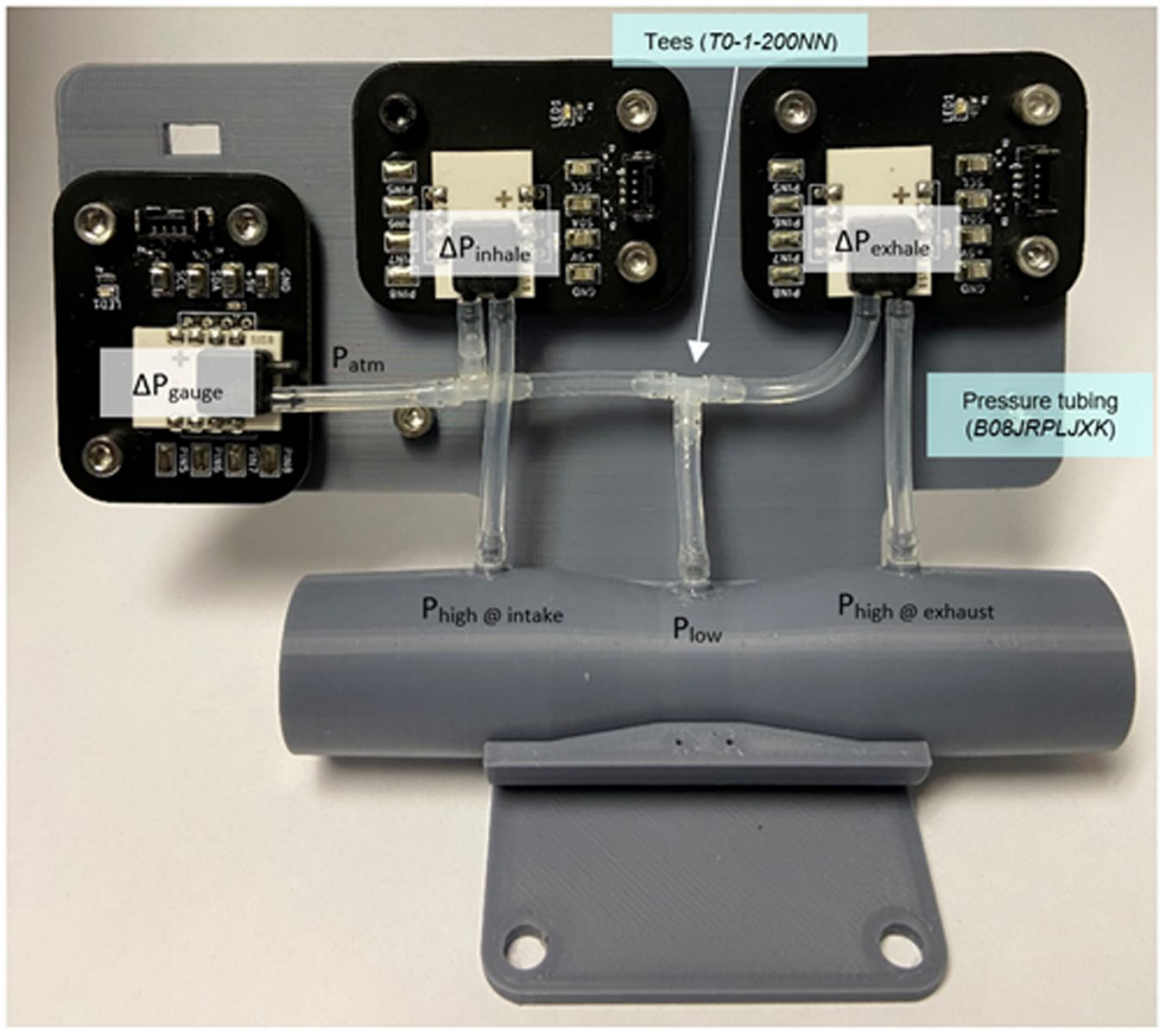
Bidirectional differential pressure sensor array^51^.

**Fig. 3:**
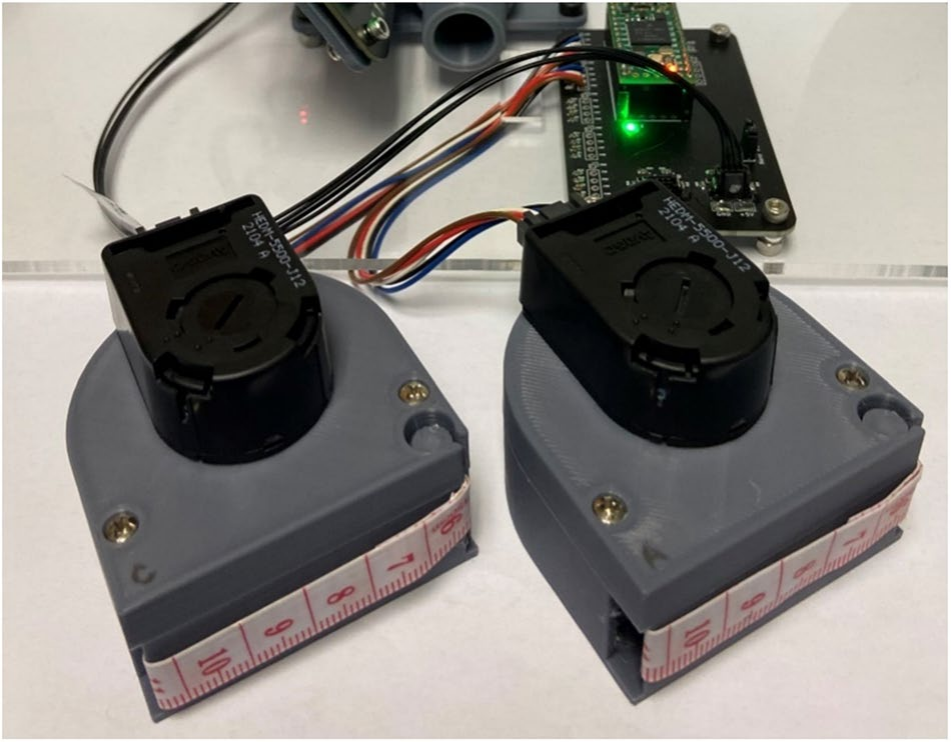
Rotary encoder dynamic circumference tape measures^51^.

Data were recorded using a custom Venturi-based bidirectional differential pressure sensor array (Fig. 2) and rotary encoder-based dynamic circumference tapes (Fig. 3). Details of the design and validation of these open-design measurement devices, including links to open-access design files, is available at Mendeley Data^51^. Data was collected using Matlab (Matlab 2021b, The Mathworks Inc, Natick, MA, USA), sampling at 100 Hz. The differential pressure over the venturi constriction was recorded in both directions and between the throat and atmosphere (gauge) (Fig. 2), alongside thoracic and abdominal circumferences (Fig. 3).

### Data processing

Data was processed in MATLAB, with both raw and processed data available in the repository. Differential pressures were converted to cmH_2_O units based on datasheet transfer function information, defined:

1 $$\Delta P\left[cm{H}_{2}O\right]=25.4\frac{Output-1638}{14745-1638}$$

The differential pressure sensors (P1J-10-AX16PA) had a pressure range of 0–10“H_2_O (0–25.4 cmH2O), corresponding to a digital count of 1638 at 0% Pressure (10% of 2^14 counts) and 14745 at 100% Pressure (90% of 2^14 counts).

The venturi differential pressures were combined, to read the differential pressure over the intake with respect to the direction of flow, and used to compute the flow:

2 $$Q={c}_{d}{A}_{2}\sqrt{\frac{2\left(\Delta P\right)}{\rho \left(1-\left(\frac{{A}_{1}}{{A}_{2}}\right)\right)}}$$

Where, A_1_ and A_2_ are cross-sectional areas at the intake and constriction, respectively (d_1_ = 15 mm and d_2_ = 10 mm). Given a discharge coefficient, c_d_ = 0.97, and the density of air, ρ = 1.225 kg/m^3^. Inspiratory start points were identified, and tidal volumes, V [L] were computed as the integral of flow, Q [L/s], over time, t [s], which is zeroed at each inspiratory start index.

Circumferences were measured using rotary encoders measuring revolutions of a tape barrel as the tape was unspooled and spooled during expansion and contraction, respectively, of the chest or abdomen^51^. Tapes captured the initial circumference during the unspooling of the tape to fit the subject^51^. Circumferences (C) were converted from rotary counts based on the initial unspooled length (C_0_ = 108 mm) and encoder counts per revolution (4096)^51^, yielding:

3 $$C={C}_{0}+2\pi r\frac{Output}{4096}$$

Given the tape barrel radius (r), calculated from the number of complete barrel revolutions, the initial radius of the full spool (r_0_ = 22 mm), and tape thickness (t_t_ = 0.15 mm):

4 $$r={r}_{0}-{t}_{t}\ast floor\left(\frac{Output}{4096}\right)$$

**Fig. 4:**
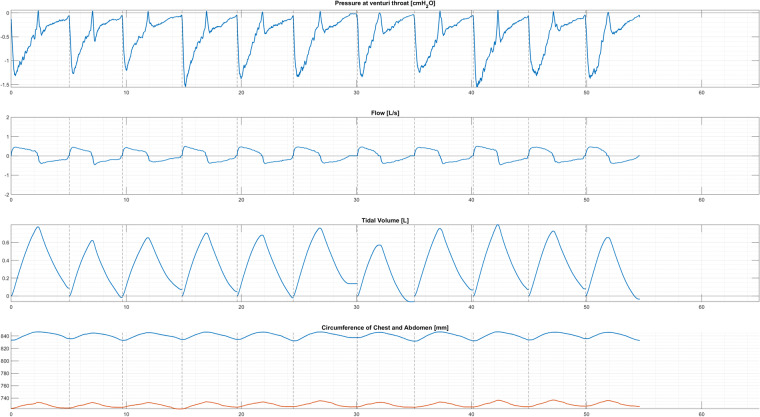
Example plotted dataset against time [s], with dashed vertical lines at identified start of inspiration indices. Subject 03 Normal breathing at ZEEP^52^.

Ultimately, data processed yielded datasets for each trial (subject, PEEP, breath type) of time [s], Gauge pressure [cmH_2_O], Flow [L/s], Tidal Volume [L], Chest circumference [mm], Abdominal circumference [mm], alongside index values for the start of inspiration (InspInd). Breaths were identified to start at the inspiratory start indices, by identification of a change in flow direction from expiratory to inspiratory, and considered to end at the point prior to the next inspiratory start index. An example of a processed dataset in Fig. 4, against time [s] with inspiratory start indexes indicated with vertical dashed lines^52^.

## Data Records

The repository contains deidentified data collected from N = 30 healthy subjects, aged 19–37 years, recruited via advertisement at the University of Canterbury^52^. Ethical consent for the trial was granted by the Human Research Ethics Committee (HREC) at the University of Canterbury (HEC 2020/14/LR). The trial included an even split of sex (15 male, 15 female), and self-identified smokers, vapers, and asthmatics were included. Subject demographic data is included in a spreadsheet (sex, height, weight, age, smoking history, history of asthma, and whether any of the trials made it feel harder or easier to breathe). A ‘*README.txt*’ file is included, which comprehensively describes file structure, naming, and contents of files in this dataset. Physionet repository data files/folders are outlined in Table 2.

**Table 2:** Dataset files^52^.

Name of data file/data set	Description
*processed-data.zip*	Processed datasets are saved as csv files (e.g. *‘CPAP2022_ProcessedData_Subject1_0cmH2O_deep.csv’*).
*raw-data.zip*	Raw datasets are saved as csv files, both processed into relevant units (e.g. *‘CPAP2022_Subject1_0cmH2O_deep.csv’*), and as raw ADC outputs (e.g. *‘CPAP2022_Subject1_0cmH2O_deep_raw.csv’*).
Figure1*.png*	Plotted Dataset for Subject 3 breathing normally at ZEEP
*Figure2.png*	Plotted Dataset for Subject 3 panting at ZEEP
*Figure3.png*	Plotted Dataset for Subject 3 breathing deeply at ZEEP
*LICENSE.txt*	Data licence (Creative commons attribution 4.0 international public license).
*README.txt*	Data descriptor file containing an explanation of file organisation and contents.
*subject-info.csv*	Spreadsheet of self-reported medical information for the 30 subjects, classified by subject number.
*code.zip*	Contains MATLAB figure (e.g. *Figure1.png*, Figure2*.png*, and *Figure3.png*) generation code (‘*FigureGenerationCode.m’*)

Processed data is saved in ‘*processed-data.zip’* in folders by PEEP setting (0, 4, or 8) and breath type (norm, pant, or deep) e.g. ‘*0cmH2O_deep*’ (Table 2). Hence, each folder contains a data file for each Subject Number (PEEP and breath type is also referenced). Data files contain pressure [cmH2O], flow [L/s], tidal volume [L], inspiratory start point indices, chest circumference [mm], and abdominal circumference [mm] against time [s]. Processed data can be plotted using the ‘*FigureGenerationCode.m*’ (Table 2). Examples of plotted data are included as ‘*Figure1.png’*, ‘*Figure2.png*’, and ‘*Figure3.png*’ for subject 3 breathing normally, panting and breathing deeply, respectively, at ZEEP (Table 2).

Raw datasets are also included as ADC direct output files and as files with processed units but differential pressures unresolved into the bidirectional flow (Table 2). Files are saved in *‘raw-data.zip’* under folders by subject number (‘01’ through to ‘30’). Files are saved in these folders by PEEP setting and breath rate type (Subject Number is also referenced).

Demographic data is included in a spreadsheet ‘subject-info.csv’ (Table 2). Spreadsheet columns A to M, respectively, contain subject number, sex (M/F), height [cm], weight [kg], age [years], history of smoking and/or vaping (Yes/No), smoking/vaping frequency (units included in column values), duration of smoking (units included in column value), history of asthma (Yes/No), asthma medication name (if applicable), frequency of use of asthma medication (if applicable, units included in column value), any trials the subject perceived to make it harder to breathe (PEEP setting [cmH2O]), and any trials the subject perceived to make it easier to breathe (PEEP setting [cmH2O]).

## Technical Validation

The hardware system validation is outlined^51^. Flow was calibrated against a TSI 4000 Series externally calibrated flow sensor^51^. Tape extension accuracy was assessed by extensions to a series of known values between 200 and 1500 mm^51^.

## Usage Notes

A prevalent issue in all PAP and especially CPAP therapy is mask leakage^53–55^. Efforts were made to reduce leakage in this trial by ensuring the mask fit very tightly at the start of the test and asking the patient to signal to the researchers if they feel any leaks around this seal. However, during preliminary analysis, volume deficits indicative of mask leakage were noted in the data from some trials. Mask leakage is a consideration when using data to identify expiratory patient-specific lung mechanics by model-based methods. Uncaptured, leakage flow may impact model parameters identified during expiratory periods and thus the accuracy of model-fit to the breath. Expiratory mask leakage affect only measured expired flow (hence volume), and so peak tidal volumes and inspiratory volumes and flow profiles were unaffected. Dynamic circumference data was captured independently, and not impacted by mask leakage.

It should also be noted gauge pressure measurements hit sensor maximum in some trials during high flow rates, predominantly seen in panting. This issue did not impact other measurements in the trial. However, in the application of models to this dataset, regions of saturated peak pressures should be considered and not be used to identify pulmonary mechanics parameters. In future trials, the gauge pressure sensor port will be relocated from the constriction and higher-range sensors implemented.

## Data Availability

Data was collected using MATLAB code and custom hardware. Design files and code are available open-access, with manufacture and usage instructions published in a HardwareX article^51^. The collected data, subject demographic information, and figure generation code are published in a Physionet repository^52^.
